# Ectopic Cerebellar Cell Migration Causes Maldevelopment of Purkinje Cells and Abnormal Motor Behaviour in *Cxcr4* Null Mice

**DOI:** 10.1371/journal.pone.0086471

**Published:** 2014-02-07

**Authors:** Guo-Jen Huang, Andrew Edwards, Cheng-Yu Tsai, Yi-Shin Lee, Lei Peng, Takumi Era, Yoshio Hirabayashi, Ching-Yen Tsai, Shin-Ichi Nishikawa, Yoichiro Iwakura, Shu-Jen Chen, Jonathan Flint

**Affiliations:** 1 Department and Graduate Institute of Biomedical Sciences, College of Medicine, Chang Gung University, Tao-Yuan, Taiwan; 2 Healthy Aging Research Center, Chang Gung University, Tao-Yuan, Taiwan; 3 The Wellcome Trust Centre for Human Genetics, University of Oxford, Oxford, United Kingdom; 4 Department of Cell Modulation, Institute of Molecular Embryology and Genetics, Kumamoto University, Kumamoto, Japan; 5 Laboratory for Molecular Membrane Neuroscience, Brain Science Institute, RIKEN, Wako, Saitama, Japan; 6 Institute of Molecular Biology, Academia Sinica, Taipei, Taiwan; 7 Center for Developmental Biology, RIKEN, Kobe, Japan; 8 Research Institute for Biomedical Sciences, Tokyo University of Science, Noda, Chiba, Japan; Institut de la Vision, France

## Abstract

SDF-1/CXCR4 signalling plays an important role in neuronal cell migration and brain development. However, the impact of CXCR4 deficiency in the postnatal mouse brain is still poorly understood. Here, we demonstrate the importance of CXCR4 on cerebellar development and motor behaviour by conditional inactivation of *Cxcr4* in the central nervous system. We found CXCR4 plays a key role in cerebellar development. Its loss leads to defects in Purkinje cell dentritogenesis and axonal projection *in vivo* but not in cell culture. Transcriptome analysis revealed the most significantly affected pathways in the *Cxcr4* deficient developing cerebellum are involved in extra cellular matrix receptor interactions and focal adhesion. Consistent with functional impairment of the cerebellum, *Cxcr4* knockout mice have poor coordination and balance performance in skilled motor tests. Together, these results suggest ectopic the migration of granule cells impairs development of Purkinje cells, causes gross cerebellar anatomical disruption and leads to behavioural motor defects in *Cxcr4* null mice.

## Introduction

CXC chemokine receptor 4 (CXCR4) is a seven-transmembrane G-protein-coupled receptor. It acts as a receptor for CXC chemokine stromal cell derived factor-1 (SDF-1, also called CXCL12). It is widely expressed in a variety of tissue types but is predominantly expressed by immune cells and in the brain. While the immune function of CXCR4 has been much studied, little is known about its role in the brain.

During embryonic mouse brain development, *Cxcr4* is expressed in ventricular zones. These are sites of stem cell proliferation. In late embryonic stages, *Cxcr4* is expressed in the hippocampus and cerebellum [1]. Embryonic data (E18.5 and P0) from *Cxcr4* knockout (KO) mice show that the cerebellum develops abnormally with an irregular external granule cell layer (EGL) and ectopically located Purkinje cells [2], [3]. These studies imply that defects in SDF-1/CXCR4 signaling result in premature migration from the EGL during embryonic cerebellar development. Indeed, SDF-1 has been shown to function as a chemoattractant and is secreted from the meninges. It attracts embryonic but not postnatal cerebellar EGL cells [4]. In SDF-1 KO mice at E15.5, premature granule cells have been detected migrating into the cerebellar anlage [5].

*Cxcr4* is highly expressed from E18.5 to P4 in the cerebellum. Subsequently, expression becomes very low or non-detectable at P14 (according to the Allen Brain Atlas [6]). Currently, the effect of CXCR4 deficiency in postnatal cerebellar development is poorly understood. This is because *Cxcr4* KO mice are embryonic lethal as a result of defects in cardiogenesis and hematopoiesis [3]. To date there has been no study into postnatal cerebellar development in CXCR4 KOs since the work of Zou *et al.* in 1998. Consequently, in order to study postnatal development and its impact on function we conditionally inactivated *Cxcr4* in the central nervous system (CNS). We here report the functional characterization of conditional inactivation of *Cxcr4* in postnatal cerebellar development.

## Materials and Methods

### Ethics Statement

All experiments were carried out in strict accordance with the recommendations in the Guide for Laboratory Animals Facilities and Care as promulgated by the Council of Agriculture. Executive Yuan, ROC. The protocol was approved by the Institional Animal Care and Use Committee of Chang Gung University (Permit Number: CGU11-007). In this protocol, all efforts were made to minimize suffering.

### Animals

*Cxcr4*(^flox/flox^) mice [7] and *Sox1-Cre* mice (Acc. No. [CDB0525K], http://www.cdb.riken.jp/arg/mutant%20mice%20list.html) [8] have been described previously and were genotyped accordingly. Rosa26-EGFP mice were purchased from National Laboratory Animal Center, Taiwan. Mice were maintained in specific pathogen-free conditions. They were housed in a 12∶12 hour light dark cycle at temperature of 22°C and a humidity level of 60–70%. Animals had ad libitum access to food and water.

### Immunohistochemistry and *in situ* hybridization

Tissue was fixed in 4% paraformaldehyde. All sections for immunohistochemistry and *in situ* hybridzation were cut to a thickness of 40 µm on a sliding microtome. For antibody staining, sections were mounted on superfrost electrostatic slides and dried overnight. Subsequently, slides were incubated in the 0.01 mol/L citric buffer for 15 min at 90°C, 3% H_2_O_2_ for 10 min, rinsed in PBS, and incubated overnight at room temperature. BrdU (Accurate, 1∶250), NeuroD (Santa Cruz, 1∶1000), Calbindin (Sigma, 1∶1000), Cleaved Caspase-3 (Cell Signaling, 1∶150) antibodies were used. Next day, following the ABC kit procedure (Vector Lab), slides were reacted with a Sigma DAB tablet. Sections were then cover-slipped with DPX. For immunofluorescence staining, sections were mounted on slides and dried overnight. On the following day, slides were incubated in the 0.01 mol/L citric buffer for 15 min at 90°C, rinsed in PBS, and incubated overnight in primary antibody solution. Pax6 (Millipore 1∶1000), GFAP (Sigma, 1∶2000), Calbindin (Sigma, 1∶1000), CNPase (Abcam 1∶1000), and NeuN (Millipore, 1∶400) antibody concentrations were used. Subsequently, slides were rinsed in PBS and incubated with Alex Fluor 568 and Alex Fluor 488 secondary antibody (1∶200) for one hour. Slides were then cover-slipped. For *in situ* hybridization, CXCR4 DIG-labeled probe was synthesized using a forward primer (ATGGAACCGATCAGTGTGAGTA) and reverse primer (ATGCTCTCGAAGTCACATCCTT). Slides were incubated in Proteinase K (Invitrogen, 20 ug/ml) for 10 min, acetylated by acetic anhydride, incubated with *Cxcr4* probe in 50% formamide at 58°C overnight. Next day, washing with SSC and MABS, then slides were incubated with anti-DIG antibody (Roche, 1∶5000) overnight. Signal was detected by NBT and BCIP for 12 hours.

### Cell counting for Granule and Purkinje cells

Brain tissue for granule and Purkinje cells counting was cut to a thickness of 20 µm on a sliding microtome. One in eight representative sections was then stained for Pax6 or Calbindin using antibodies. Four sagittal sections close to the middle of the cerebellum were selected for cell counting. For Purkinje cells counting, the total number of cells present on the four sections was counted. For granule cells counting, we selected 24 areas (100×200 µm^2^) inside the granule cell layer and counted the number of granule cell in each square using Image J.

### Golgi staining

Brains were harvested and stained using an FD rapid Golgistain™ Kit in accordance with the manufacturer's instructions (FD Neuro Technologies). Brains were sectioned sagittally at a thickness of 120 µm using a microtome. The numbers of terminal dendrites of Purkinje cells were counted. A z-series of images were taken of purkinje cells and a focused image produced via photo-merge stacking in Adobe Photoshop CS5.

### mRNA quantitification

P1 cerebella were collect from WT and KO mice. mRNA was extracted using a RNeasy Lipid Tissue Kit (Qiagen). cDNA was then made using SuperScript III reverse transcriptase (Invitrogen). Expression was measured by quantitative PCR using the following primers: *Cxcr4* (F: GACTGGCATAGTCGGCAATG, R: AGAAGGGGAGTGTGATGACA AA) *Neurog2* (F: AACTCCACGTCCCCATACAG, R: TGCCAGTAGTCCACGTCTGA) *Ptf1a* (F: CTGCGACAAGCCGCTAATG, R: GAAGGCGTCGTTGATGGACT). β-actin (F: CATCACTATTGGCAACGAG, R: GGCATAGAGGTCTTTACGG). Experiments were performed in duplicate. Gene expression levels were calculated by the ΔΔC_t_ method and normalized against a β-actin control.

### Cerebellum culture and staining

The cerebellum cell culture method was modified from a previous study [9]. Cerebella were dissected from WT or KO E17 embryos. They were then immersed in 12 ml of ice-cold HBSS in a 15 ml falcon tube. The medium was then discarded and the cerebella placed in 1 ml of HBSS supplemented trypsin (0.1%) and heated for 10 min at 37°C in the water bath. 1 ml of ice-cold Normal horse serum and 50 µl of 1% DNase was then added. The media was pipetted gently to dissociate cells. 1×10^6^ cells per well in 90 µl were plated onto glass cover slips (12 mm in diameter) coated with poly-D-lysine and placed in a humidified CO_2_ incubator (5% CO_2_ at 37°C). One milliliter of culture medium was added to each well after 3 hours. The medium was composed of DMEM-nutrient mixture of Ham's F-12 supplemented with insulin (7.5 µg/ml), transferrin (75 mg/ml), progesterone (15 nM), sodium selenite (22.5 nM), GlutaMAX (3 mM), triodothyronine (0.4 ng/ml), gentamicin (7.5 µg/ml), and putrescine (75 µM). For the CXCR4 antagonist experiment, AMD3100 (a gift from Dr. Kak-Shan Shia, NHRI, Taiwan) was added to the culture wells accordingly (0, 2.5 µg/ml, 10 µg/ml). The cerebellar cultures at 21 DIV were fixed with 4% paraformaldehyde in PBS for 15 minutes at room temperature. The cultures were incubated with calbindin antibody (Sigma, 1∶1000) and beta-tubulin (abcam, 1∶1000) in 0.5% Triton X-100 and 5% serum in PBS for 2 hours at room temperature. They were then incubated with fluorescein-labeled secondary antibodies (1∶200) for 1 hour at room temperature. The numbers of terminal dendrites of Purkinje cells were counted.

### Microarray analysis

Gene expression analysis was performed with multiple biological replicates for WT (n = 8) and *Cxcr4* KO (n = 7). RNA was prepared from cerebella from one-day-old WT and *Cxcr4* KO mice. RNA was reverse transcribed into cRNA and biotin-UTP labeled using the Illumina TotalPrep RNA amplification kit (Ambion) and hybridized to the Illumina mouse Rsfseq-8v2 Expression BeadChips. Image data were extracted using the Illumina GenomeStudio software. Raw expression values were log2 transformed and median normalized. The expression array data is available on Gene Expression Omnibus (GSE48788).

### Pathway enrichment analysis

For pathway enrichment analysis, the normalized data of entire 17925 transcripts were uploaded to the GSEA software (v 2.0, http://www.broad.mit.edu/gsea/). Signal-to-noise ratio was used to generate the ranked list for all genes. The association between a given gene set and a treatment group was measured by the non-parametric running sum statistic termed the enrichment score (ES).The gene sets used are from Molecular Signatures Database (MsigDB), catalog C2 functional sets, subcatalog canonical pathways, which include 241 gene sets from pathway databases (version 2.5, updated by April, 2008). These gene sets are canonical representations of 241 biological process compiled by KEGG. The cut-off p-values were assigned based upon 1000 random permutation tests. The normalized enrichment score (NES) was calculated based on the size of the gene set and its enrichment score. The nominal p-value was calculated after permutation testing of the microarray samples. The false discovery rate (FDR) was calculated to correct for multiple hypothesis testing. As recommended by the software developer, a gene set is considered significantly enriched when its p-value is less than 0.05 or FDR score is less than 0.25.

### Heatmap and hierarchical clustering

All genes in the leading edge and the trailing edge of the eight significantly altered pathways listed in Table S1 were selected for hierarchical clustering analysis. A total of 69 genes were included in the analysis. Two-dimensional hierarchical clustering was performed by Ward's linkage method using Pearson's correlation as a measure of similarity. Intensity on the heatmap represents standardized expression level of individual gene in each sample. Hierarchical clustering analysis and intensity standardization were performed using Partek Genomics Suite (v 6.4).

### Behavioral tests

#### Activity box

Mice were housed separately in transparent plastic cages for two hours. Two laser beams were fired across opposite ends of the cage. If a mouse positioned itself between the beam emitter and sensor, a “beam break” would be automatically counted by a computer. If a mouse broke both beams in succession, thereby transversing the length of the cage, a “crossover” was counted. The counts for beam breaks and crossovers were measured over 24×5 minute intervals.

#### Inverted mesh grid grip test

Mice were placed on the centre of a 43 cm square wire mesh grid consisting of 12 mm squares of 1 mm diameter wire. The grid was surrounded by a 4 cm thick wooden frame which prevented mice climbing to the converse side. Once a mouse was positioned, the grid was turned upside down and elevated in order to force the mouse to grip the wire to avoid falling. The time taken for a mouse to fall was recorded. If an animal did not fall, the experiment was concluded after one minute.

#### Grip strength test

We followed the manufacturer's instruction for the Grip Strength Meter (UGO Basile). Mice were placed over a base plate, in front of a grasping bar. The bar was fitted to a force transducer connected to the peak amplifier. Mice were pulled by the tail, and the mice grasp the bar. Within 20 seconds, maximal grip force was measured.

#### Accelerating rotarod

Mice were held by the tail and placed on the rotarod, facing away from the direction of rotation. The rotarod moved at an initial speed of 4 rpm. After 10 seconds, the rod speed was accelerated at a rate of 20 rpm per minute. Once acceleration had been triggered, the time taken for mice to fall was noted.

#### Gait analysis

When mice spontaneously walked at a velocity of 20 cm/sec their motion was recorded by BCamCapture Version (Clever Sys Inc). TreadScan (Clever Sys Inc) was then used to analysis the standing and swing phases during gait cycles. The stand time is the time elapsed while the foot is in contact with the tread, in its stance phase. The swing time is the time elapsed while the foot is in the air, in its swing phase.

#### Ladder Rung Walking Test

Mice were placed on a Ladder Rung walking test apparatus. Mice were trained to walk through an irregular rung arrangement. Mice were given three trials in total, and the number of missing steps counted. The ladder rung task apparatus was modified from the published version for mice [10].

### Behavioral statistics

The activity box data was analyzed via a paired T test, with KO results analyzed against their WT counterparts for a given time bin. The inverted mesh test was analyzed via a survival analyzed test. T tests were used to analysis grip strength, rotarod results, gait analysis and ladder rung walking. Values represent means ± SEM. All stats were performed on Graphpad Prism version 5.

## Results

### CXCR4 inactivation causes granule cell ectopia and agenesis of the cerebellar folia

**Figure 1:**
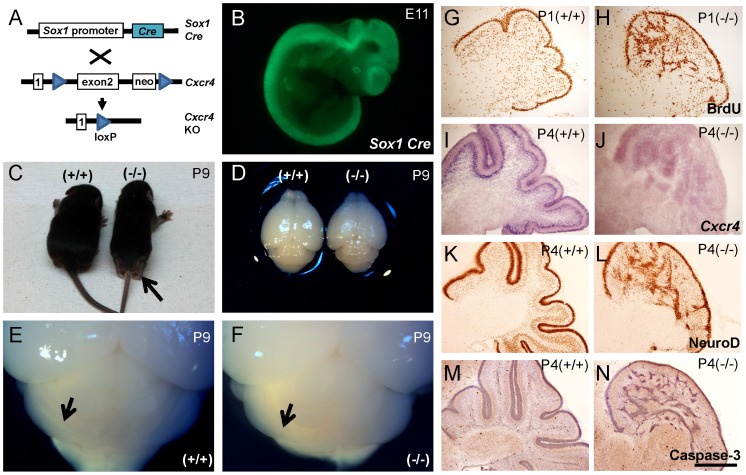
CNS specific *Cxcr4* inactivation causes cerebellar abnormalities. (A) *Cxcr4* floxed mice were crossed with *Sox1-Cre* to generate CNS specific *Cxcr4-*deficient mice. (B) *Sox1-Cre*; *Rosa26*-eGFP reporter mice demonstrate robust Cre recombinase activity in the Central Nerve System of E11 embryos (C) Approximately half of *Cxcr4* KO pups showed a dragging phenotype in their hind limb prior to weaning. (D) *Cxcr4* KO mice had smaller, lighter brains (n = 4). (E,F) Unlike WT, KO mice did not develop obvious cerebellar folia. (G,H) Two hours of BrdU labeling in P1 KO mice revealed that the proliferating external granule cells invaded the cerebellar anlage. (I,J) In P4 cerebella, *Cxcr4* expression is mainly in external granule cell layer and Purkinje cell layer with some expression also present in the granule cells layer. (K,L) NeuroD antibody staining shows that KO mice had a disorganized granule cells layer. (M,N) There was no obvious increased cleaved caspase-3 staining detected in the KO cerebellum. Values represent means ± SEM. Scale bar = 500 µm in G-N. (**p<0.01).

Mice lacking CXCR4 in the CNS were generated by crossing mice harboring loxP sites flanking exon 2 of the *Cxcr4* gene (*Cxcr4^flox/flox^*) [7] with *Sox1-Cre* mice [8] (Fig. 1A). *Sox1* expression defines the neuronal precursors of the embryonic central nervous system [11], [12]. *Sox1-Cre* mice express Cre throughout the neural tube at E9.5 [8]. To check the specificity of this *Sox1-Cre* expression, we crossed *Sox1-Cre* mice with Rosa26-EGFP reporter mice. We showed that the Cre recombinase is expressed in the neural tube at E11 (Fig. 1B). In our study, we used *Cxcr4^flox/flox^*; *Sox1-Cre* mice as KO mutants and the littermates without Cre were used as wild-type (WT) controls. KO mice were viable and fertile. Prior to weaning, approximately half of KO pups (*Cxcr4^flox/flox^*; *Sox1-Cre*) were observed to feature a limping gait phenotype with dragging of their hind limbs (Fig. 1C). From our observations, this limb dragging phenotype became less severe after weaning age. Mice were sacrificed at P9, and KO brains were found to be smaller in size and significantly reduced in weight compared with WT littermates (*p* = 0.0064, n = 4) (Fig. 1D). On macroscopic examination, KO mice were also found to feature a smaller cerebellum which lacked the well-defined cerebellar folia observable in the WT mice (Fig. 1E,F).

We further characterized the abnormalities of the cerebellar folia by injecting BrdU (50 mg/kg, i.p.) into P1 animals. We sacrificed them two hours later to label the proliferating cells. The BrdU imaging showed a subset of proliferating granule cells of the EGL invading the cerebellar anlage (Fig. 1G,H). *Cxcr4* expression in the cerebellum is greatest between P3 to P5 [13]. We used in situ hybridization to show *Cxcr4* expression was very low in our P4 KO mice whilst abundant in WT, particularly in the Purkinje cells layer and EGL (Fig. 1I,J). The expression pattern of *NeuroD*, a transcription factor required for differentiation of granule cells [14], showed that WT littermates featured a well-organized granule cell layer (GCL). In contrast, differentiating granule cells were ectopically located in the KO mice. Furthermore, no folia were formed in the KO cerebellum (Fig. 1K,L). These images suggest that knocking out *Cxcr4* not only affects the EGL, but also the GCL and Purkinje cell layers during cerebellar development. To investigate whether there was increased apoptosis in the irregularly distorted cerebellum, we stained for cleaved caspase-3. We found no evidence of an increase in the number of cleaved caspase-3 labeled cells in the P4 KO (Fig. 1M,N).

### CXCR4 effects Purkinje cell dendritogenesis in vivo, but not in vitro

**Figure 2:**
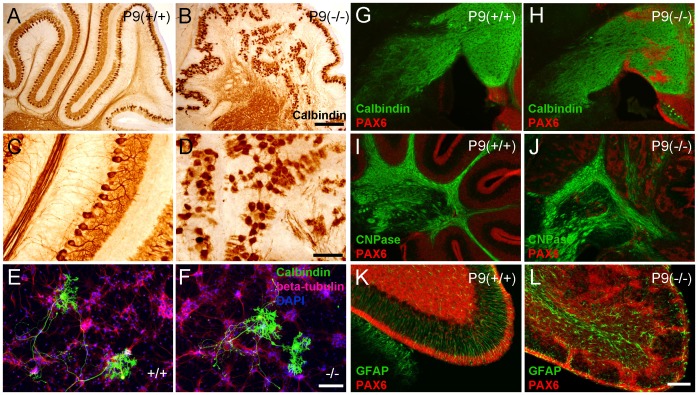
*Cxcr4* deficiency inhibits Purkinje cell dendritogenesis caused by ectopic granule cells migration in the developing brain. (A,B) Calbindin staining revealed ectopic and disorganized Purkinje cells in P9 KO mice. (C,D) A greater magnification of figure A and B shows Purkinje cells from P9 KO mice have less dendritogenesis and feature axon disorganisation (n = 6). (E,F) Cerebellum cell culture from E17 KO and WT embryos. Cabindin staining (green) shows there is no significant difference in the dendritic development of Purkinje cells between KO and WT cells (n = 12). No obvious defect in Purkinje cell axon growth was observed. (G,H) Calbindin staining reveals the axons of Purkinje cells project to the deep cerebellar nuclei correctly in KO mice. (I,J) In P9 WT mice, we observed compact myelinated axon fibers by staining CNPase. In KO mice these myelinated fibers are disorganized and sporadic. (K,L) Pax6-expressing granule cells migrate along the GFAP-positive radial glia fibers in WT mice. In KO mice, the GFAP positive cells do not align neatly in the Molecular Layer and the Pax6-expressing granule cells do not migrate appropriately. Scale bars = 300 µm in A,B; 100 µm in E,F. 200 µm in G,H,I,J. 100 µm in K,L.

We explored the development of Purkinje cells in the *Cxcr4* KO mice. The Purkinje cell marker, Calbindin, showed that Purkinje cells were ectopically located in P9 KO mice. The Purkinje cell layer was much less organized compared with WT mice (Fig. 2A,B). The greater magnification of the Calbindin images (Fig. 2C,D) revealed that whilst dendritic arborization was less expansive and axons were disorganized in KO mice these axons do project to deep cerebellar nuclei. We also observed that dendritic elaboration is reduced in Purkinje cells which have inappropriately migrated inwardly, away from the molecular layer. This suggests the ectopic migration affects dendritic development.

Thus, we sought to establish whether disrupting SDF-1/CXCR4 signaling impaired dendritogenesis in Purkinje cells directly or if the Purkinje cell defects were a consequence of ectopic migration. To answer this question, we isolated WT and KO cerebella from E17 embryos and co-cultured granule cells and Purkinje cells together. The cultured cells were in the same medium for three weeks to allow the Purkinje cells to receive factors from the granule cells and fully develop. Three weeks later, we analyzed the dendritic arboration of the Purkinje cells from KO and WT by using a Purkinje cell marker, Calbindin, and a neuronal marker, beta-tubulin class III. The results showed that there is no difference in dendritic complexity upon quantifying the number of terminal branches (*p* = 0.19, n = 12) (Fig. 2E,F). We also did not observe any obvious difference in axon development between KO and WT Purkinje cells. To confirm this result, we repeated this experiment by adding AMD3100 (0, 2.5 µg/ml, 10 µg/ml), a CXCR4 antagonist, into the cultured cells. There was no difference in dendritic complexity between the three groups (*p* = 0.85, n = 12). Both *in vitro* results indicated that the Purkinje cell defect detected in *Cxcr4* KO mice were not a direct effect of CXCR4 loss. Consequently, our findings strongly suggest that the abnormal migration and alien extracellular environment underlie the mal-development of the KO Purkinje cells *in vivo*.

### Radial glia cells, granule cell migration, and Purkinje cell projections

**Figure 3:**
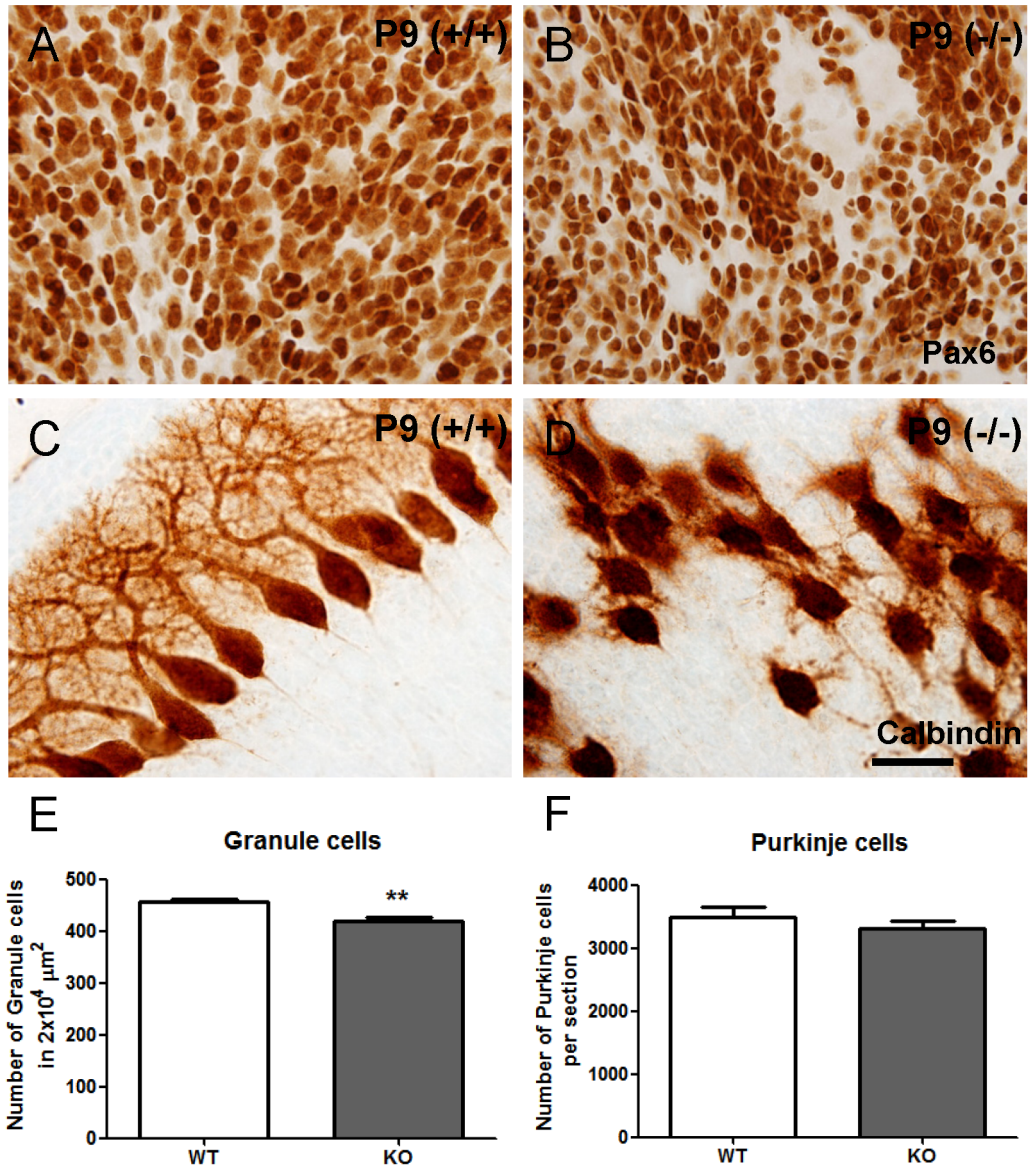
*Cxcr4* deficiency results in lower density of granule cells, but no difference in the number of Purkinje cells in P9 cerebella. (A,B) The Pax6 staining shows the granule cells in the WT and KO P9 cerebella. (C,D) The Calbindin staining shows the disorganized Purkinje cells in KO cerebellum. (E,F) There is lower density of granule cells in KO cerebella, but no difference in the number of Purkinje cells. Values represent mean ± SEM (** p<0.01). Scale bar = 30 µm in A,B,C,D.

We used Calbindin staining to analyse the projections of Purkinje cells. Surprisingly, we found that Purkinje cell axons end correctly in the deep cerebellar nuclei in KO mice (Fig. 2G,H). We also stained for the oligodendrocyte marker, CNPase. We found that whilst axons in both WT and KO mice successfully projected to deep cerebellar nuclei, the projections in KO mice were more sporadic and untidy (Fig. 2I,J). To determine whether some of these stained myelinated fibers were Purkinje cells axons, we performed a double labelling experiment for Calbindin and CNPase (Fig. S1A,B). We found a substantial amount of Calbindin staining colocalized with CNPase in both WT and KO cerebellum (Fig. S1C,D). These results suggest that the ectopically located Purkinje cells in KO mice still send myelinated axons to their targets in the deep cerebellar nuclei. Next, we studied granule cell migration using GFAP-labeled radial glia cells. In WT mice, Pax6 positive granule cells migrate along the radial glia fibers which extend to the apical surface. In KO mice, the radial glia scaffolds were not well aligned resulting in disrupted granule cell migration (Fig. 2K,L). Meanwhile, it has been demonstrated that the decrease in the number of granule cells may affect the location of Purkinje cells and the development of their dendritic trees [15]. To better understand whether there are difference in the number of granule cells and Purkinje cells in this CXCR4 KO model, we used thinner section (20 µm) for cell counting. The result show there is a significant lower density of granule cells in the KO cerebella (*p* = 0.001, n = 6) (Fig. 3A,B,E). However there is no significant difference in the number of Purkinje cells between KO and WT (*p* = 0.35, n = 6) (Fig. 3C,D,F).

### Granule cells, Purkinje cells, and axon projection in adult KO mice

**Figure 4:**
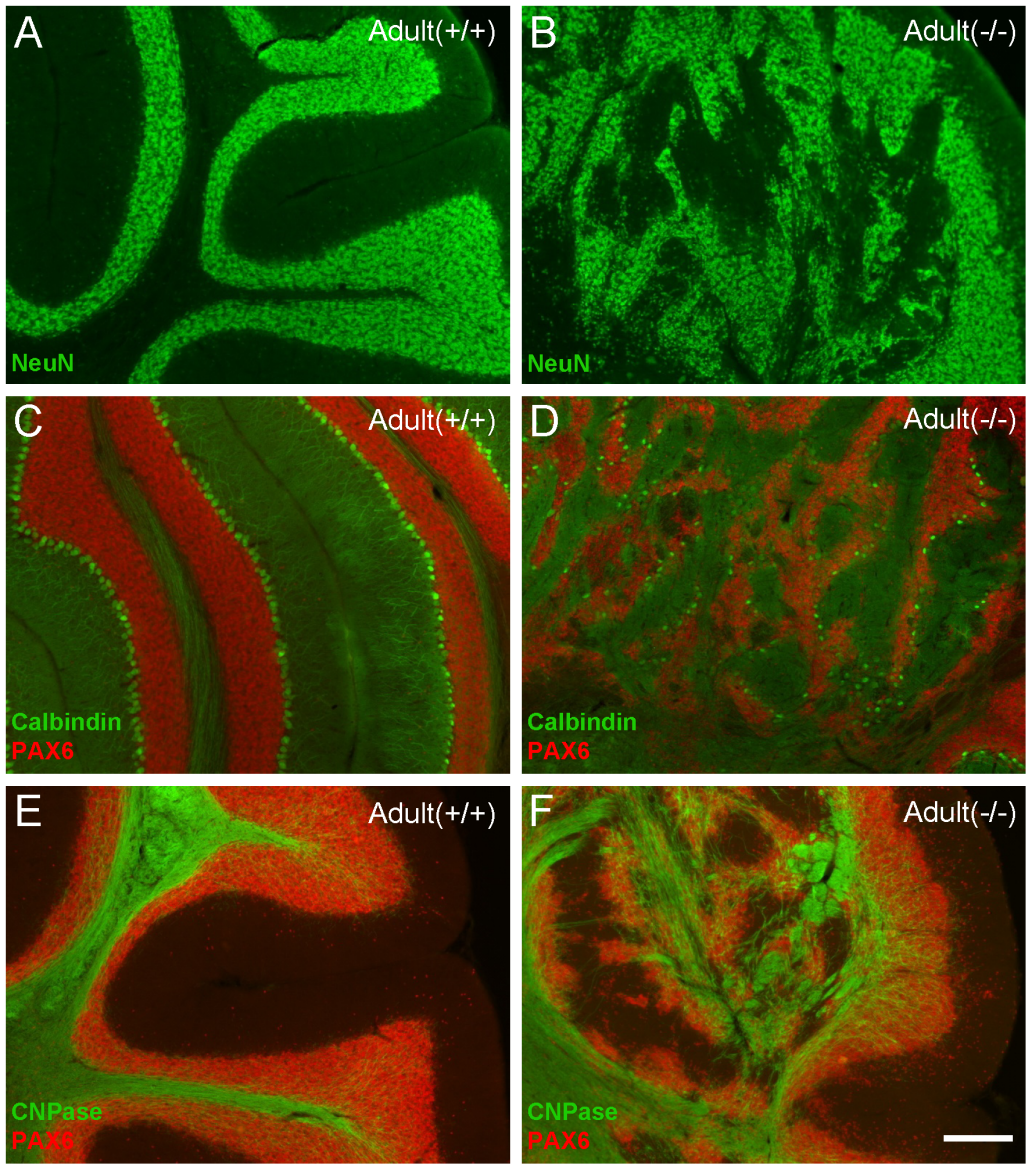
*Cxcr4* deficiency results in ectopic granule cells, Purkinje cells, and disorganized axon projections in adult mice. (A,B) The NeuN staining in adult cerebellum revealed granule cells ectopia and absent cerebellar lobules. (C.D) Calbindin staining reveals disorganized Purkinje cells and reduced dendritic elaboration. (E.F) Myelinated axon fibers, shown via a stain against CNPase, are disorderly in KO mice. Scale bar = 200 µm.

To establish if this defect was corrected during later development, and to explore the cell fate of granule and Purkinje cells, we examined the cerebellum histology in adult mice (three months old). We observed that ectopic granule cell clusters remain and the cerebellar lobules are still absent in adult KO mice (Fig. 4A,B). The Calbindin staining images revealed that the abnormally aligned Purkinje cells in KO mice remain alive but featureless dendritic elaboration than WT (Fig. 4C,D). To better understanding the development of adult Purkinje cells, we use Golgi staining to investigate the dendrites (Fig. S2A,B). KO Purkinje cells featured less complex dendritic arborization compared with WT (*p*<0.001, n = 12). Next, we investigated the axon projection by using CNPase staining. Our findings are similar to what we observed in the developmental stage: axons project to the deep cerebellar nuclei but fibers are disorganized in KO mice (Fig. 4E,F).

### Gene expression profiling of *Cxcr4* null cerebellum

To further understand the molecular changes associated with the cerebellar phenotypes, we conducted a microarray study to compare the transcriptome profile between WT and *Cxcr4* KO cerebella from P1 mice (n = 8 for WT and 7 for KO). *Cxcr4* KO animals showed a significant decrease in *Neurog2* (fold-change = −1.65, *p*<0.001) and *Ptf1a* (fold change = −1.86, *p*<0.001) gene expression levels. Recent evidence suggests that *Neurog2*, a direct downstream target of *Ptf1a*, regulates Purkinje cell dendritogenesis [16], [17]. Cerebellar GABAergic neurons are generated from *Ptf1a* expressing neuroepithelial cells. *Ptf1a* is essential for cerebellar development in mice and humans [18], [19], [20]. Therefore, to confirm our array result, we used this P1 cerebella tissue to measure the relative mRNA levels via quantitative PCR. We showed a large reduction of *Cxcr4* expression in KO mice (*p*<0.001, n = 11). There were also significant reductions in both *Ptf1a* and *Neurog2* expression (*p*<0.001, n = 11). This result implies alterations to *Ptf1a* and *Neurog2* pathways may affect the dendritogenesis of Purkinje cells in *Cxcr4* deficient mice.

**Figure 5:**
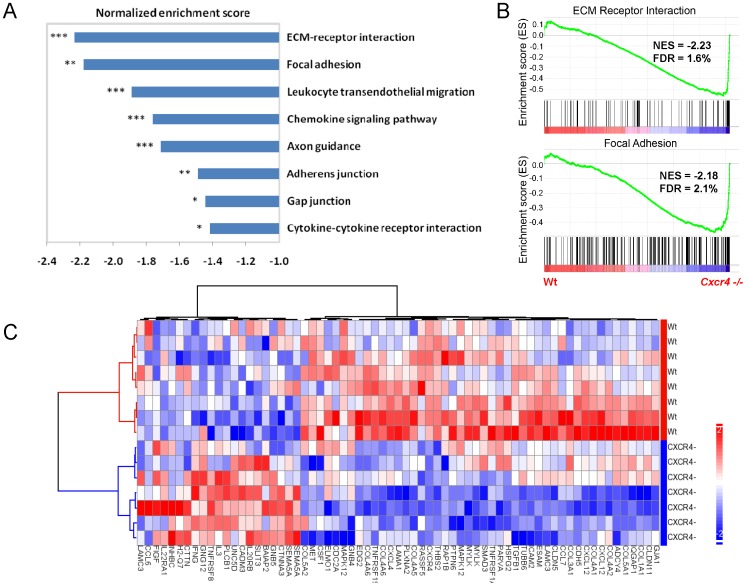
Gene set enrichment analysis identified pathways and genes significantly altered in *Cxcr4* KO mice. (A) Pathways significantly down-regulated in *Cxcr4* KO mice compared to WT mice. The normalized enrichment scores (NES) and norminal p-values are indicated for each gene set. (B) GSEA comparison of *Cxcr4* KO and wild type cerebella for enrichment or depletion of ECM-receptor interaction or focal adhesion-associated gene expression. (C) Heatmap and hierarchical clustering of 69 significantly up- and down-regulated genes from pathways shown in A. *Cxcr4* and *Cxcl12* (SDF-1) are highlighted in red. (* p<0.05, ** p<0.01, *** p<0.001).

In addition, the *Cxcr4* KO also displayed a significant decrease in *Cxcl12*, suggesting a positive regulatory loop between CXCR4 and its physiological ligand. We also performed a gene set enrichment analysis to identify biological pathways that were significantly altered (nominal *p*<0.05) (Table S1). Several biological pathways related to chemokine receptor interaction, cell mobility, and axonal guidance were found to be significantly down-regulated in C*xcr4* KO animals (Fig. 5A). The two most significantly affected pathways were the “ECM receptor interaction” and “focal adhesion” (Fig. 5B). A closer examination of differentially expressed genes in these pathways revealed that the *Cxcr4* KO is associated with an extensive decrease in genes involved in cell-cell adhesion (Fig. 5C). Together, these results indicated that a widespread inhibition of genes involved in chemotaxis, cell migration and axon guidance may underlie the phenotype observed in *Cxcr4* KO animals.

### Knocking out CXCR4 compromises the motor and coordination systems

**Figure 6:**
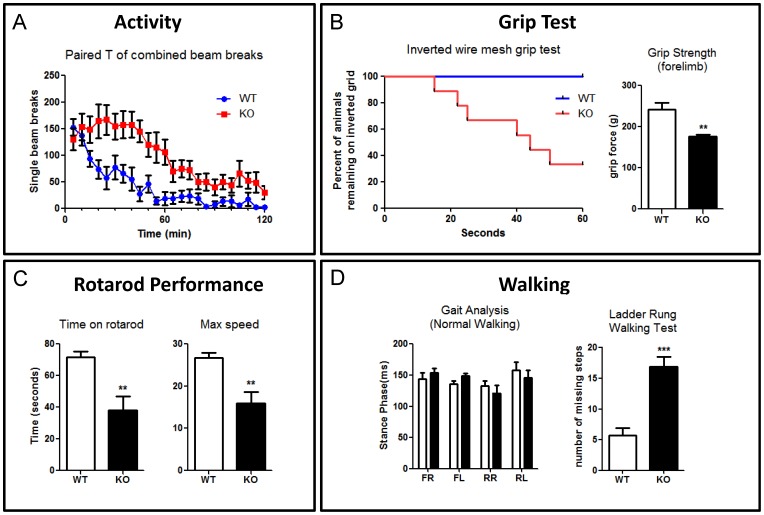
*Cxcr4* deficient mice showed poor balance and coordination. (A) KO mice have higher level of activity in 120 min locomotion test (n = 9) (B) Left: More than half of KO mice are unable to stay on an inverted wire mesh grid during a 60 sec test. All of falling mice failed to grasp the grid appropriately with their hind limbs (n = 9). Right: A pull test to measure the maximum grip strength of forelimb within 20 seconds. KO mice showed significant weaker forelimb strength (n = 6). (C) In a rotarod test (n = 9), KO mice showed poor performance and fall of faster (left) and at a slower speed (right). (D) Left: Gait analysis reveal no significant difference in stance phase between WT and KO mice in normal walking (n = 6) (FR, front right; FL, front left; RR, rear right; RL, rear left). Right: KO mice missed a significantly higher percentage of steps in the ladder rung walking test (n = 6) (**p<0.01, ***p<0.001). Values represent mean ± SEM.

Finally, to assess the effect on walking and motor behavior, we conducted a series of behavioral experiments. First, we assessed locomotor activity (Fig. 6A). In a two hour long test, KO mice showed higher activity than WT (*p*<0.001, n = 9). Next, to test muscle strength and grip, we performed an inverted mesh grid grip test. During the 60 second test, more than half of mutant mice fell (*p* = 0.0053, n = 9), while all the WT mice still remained on the inverted grid (Fig. 6B left). All of the mutant mice that fell had failed to use their hind limb to grasp. We investigated whether the tendency to fall was related to grip strength, lack of coordination, or both. We tested forelimb grip strength using a grip strength meter. KO mice had a lower forelimb grip strength (*p*<0.0033, n = 6) (Fig. 6B right), suggesting that they fell could be due to inability to grip. *Cxcr4* KO mice feature ectopic collateral branching from the corticospinal tract and this may explain this grip strength result [21]. To assess balance and coordination, we performed a rotarod test. The mutant mice fell sooner (*p* = 0.003, n = 9) and at a lower speed (*p* = 0.0023, n = 9), suggesting they also have poor balance and coordination (Fig. 6C). We then assessed normal walking and skilled walking gait of the animals. For normal walking, we utilized a gait analysis treadmill to examine the stance time and swing time. Surprisingly, there was no significant difference for stance time (n = 6) (Fig. 6D left). Skilled walking was tested using a ladder rung walking task in which the spacing of the rungs was uneven. In this task, the mutant mice made more missteps (*p*<0.001, n = 6) (Fig. 6D right). Overall, our results clearly demonstrate that motor and coordination systems, have been compromised in the *Cxcr4* KO mice.

## Discussion

The effect of CXCR4 deficiency on fetal cerebellar development has been known for more than a decade. However, study of postnatal cerebellar development has previously not been investigated. Thanks to Cre-Lox system, we were able to knock out the CXCR4 gene conditionally and bypass embryonic lethality. To our knowledge, by using a conditional KO model to bypass embryonic lethality, ours is the first study to explore the importance of *Cxcr4* in postnatal cerebellar development.

Our postnatal results are consistent with previous *Cxcr4* KO studies which demonstrated the irregular EGL and numerous granule cell clumps within the cerebellar anlage in the fetal cerebellum [2], [3]. This suggests CXCR4 is required for proliferating granule cells to detach in the EGL. In addition, we observed ectopic and abnormal development of Purkinje cells in the KO mice. Ectopic Purkinje cells had far less dendritic arborization and featured disorganized and sporadic axon projections. To establish if SDF-1/CXCR4 signaling directly influences dentritogenesis, we co-cultured Purkinje cells and granule cells from both WT and KO E17 embryos. There was no notable morphological difference between WT and KO Purkinje cells for either the CXCR4 antagonist treated or non-treated groups. CXCR4 is not required for the Purkinje cell dendritic development *in vitro*. This suggests the *in vivo* abnormalities caused by knocking out *Cxcr4* may be due to abnormal migration and a foreign extracellular environment rather than a direct effect of knocking out the gene. Additionally, we detected ectopic location and lower density of granule cells in the KO cerebella. This may contribute to abnormal development of the dendrites of Purkinje cells. Furthermore, although our cell count per section showed no difference compared to WT cerebella, since the volume of the KO cerebellum is reduced there should be a decrease in the total number of PCs in the KO cerebellum.

We went on to assess the granule and Purkinje cells in adult *Cxcr4* KO mice. A previous paper demonstrated *Cxcr4* deficient mice feature abnormal development of the dentate gyrus neonatally with distinct inner and outer blades failing to form. This hippocampal defect is corrected from P14 into adulthood [22], [23]. However, the postnatal developmental process does not correct the irregular cerebellum in adult *Cxcr4* KO mice. The abnormal cerebellum, reduced dendrite density and chaotic axon projections of Purkinje cells are still present in *Cxcr4* null mice in adulthood. However, these axon projections do successfully project to the deep cerebellar nuclei.

Thus, to look for the factors that may contribute to the cerebellar phenotype caused by *Cxcr4* deficiency, we measure the gene expression pattern change from the P1 cerebellum. The gene expression profile showed the two most significantly affected pathways were the “ECM receptor interaction” and “focal adhesion”. For example, genes encoding extracellular matrix components such as collagens (*Col1a1*, *Col1a2*, *Col2a1*, *Col3a1*, *Col4a1*, *Col4a5*, *Col4a6*, *Col5a1*, *Col5a2*, *Col6a1*, *Col6a2* and *Col11a2*) and laminins (*Lama1*, *Lama2*, and *Lama3*) were significantly reduced in KO animals. Similarly, genes encoding for signaling molecules that promote cell migration, such as *Met* and *Mylk*, were also down-modulated in KO animals. Conversely, *Slit3* and *Sema5a*, were significantly increased in knockout mice. SLIT3 is a negative regulator for CXCR4 and CXCL12 and inhibits neurite outgrowth in cultured embryonic and fetal stem cells [24], [25]. SEMA5A inhibits glioma cell migration through RAC1 inactivation [26]. These results further support the hypothesis that a cell migration defect results in extra cellular environment unsuitable for the maturation of developing Purkinje cells. Recently, it has been suggested that Neurog2 is a key regulator of Purkinje cell development and maturation [17]. Ptf1a is a direct regulator of Neurog2 and is also required for the specific generation of Purkinje cells [16], [27]. In our expression array, we detected lower level of expression of these two genes in the KO. We confirmed this finding with quantitative PCR. This suggested that a lower level of *Neurog2* and *Ptf1a* may partially contribute to the defect of Purkinje cell dentritogenesis.

Finally, we assessed the motor and coordination systems of the KO mice. Historical evidence shows that the cerebellum is heavily involved in regulating these systems and that the behavioral defects observed in the KO are consistent with the cerebellar and Purkinje cell abnormalities we observed histologically [28], [29], [30]. Not surprisingly, the KO mice performed poorly in coordination, muscle strength, balance, and skilled walking tests.

In summary, we used *Cxcr4* conditional KO mice to investigate the role of CXCR4 in postnatal cerebellar development, transcriptome profile, and motor behavior. We have provided a systematic study of the effects of knocking out *Cxcr4* within the cerebellum from early postnatal to adult life. We found that CXCR4 is essential for cell migration and that the ectopic positioning of granule cells causes the Purkinje cell ectopia and aberrant dendritogenesis. Our data suggests that Purkinje cells develop abnormally *in vivo* in *Cxcr4* null mice due to an altered cerebellar environment resulting from changes to ECM receptor interactions and focal adhesion. In a series of behavioral tests, the KO mice exhibit poor performance in balance, coordination and skilled walking behavior. This is consistent with functional disruption of the cerebellum. In this study, we provide data on postnatal phenotypes to demonstrate that failure of CXCR4 mediated signaling during early development has profound implications for the development of the cerebellum throughout life.

## Supporting Information

Figure S1 **(A,B) The CNPase and Calbindin staining shows that Purkinje cells send axons to deep cerebellar nuclei in both WT and KO mice.** (C,D) Greater magnification reveals these Purkinje cell axons are myelinated in both WT and KO mice. Scale bars = 200 µm in A,B; 50 µm in C,D.
(TIFF)

Figure S2 **(A,B) Golgi staining showed Purkinje cells in adult KO mice have significant less complex dendritic arborisation. Scale bars = 100 µm.**
(TIF)

Table S1 **Significantly altered pathways in Cxcr4 vs wild-type comparison.**
(XLSX)

## References

[1] Tissir F, Wang CE, Goffinet AM (2004) Expression of the chemokine receptor Cxcr4 mRNA during mouse brain development. Brain Res Dev Brain Res 149: 63–71. 15013630 10.1016/j.devbrainres.2004.01.002

[2] Ma Q, Jones D, Borghesani PR, Segal RA, Nagasawa T, et al (1998) Impaired B-lymphopoiesis, myelopoiesis, and derailed cerebellar neuron migration in CXCR4- and SDF-1-deficient mice. Proc Natl Acad Sci U S A 95: 9448–9453. 9689100 10.1073/pnas.95.16.9448 PMC21358

[3] Zou YR, Kottmann AH, Kuroda M, Taniuchi I, Littman DR (1998) Function of the chemokine receptor CXCR4 in haematopoiesis and in cerebellar development. Nature 393: 595–599. 9634238 10.1038/31269

[4] Zhu Y, Yu T, Zhang XC, Nagasawa T, Wu JY, et al (2002) Role of the chemokine SDF-1 as the meningeal attractant for embryonic cerebellar neurons. Nat Neurosci 5: 719–720. 12080344 10.1038/nn881 PMC2072873

[5] Yu T, Huang H, Li HF (2010) Stromal cell-derived factor-1 promotes migration of cells from the upper rhombic lip in cerebellar development. J Neurosci Res 88: 2775–2786. 20568288 10.1002/jnr.22454

[6] Lein ES, Hawrylycz MJ, Ao N, Ayres M, Bensinger A, et al (2007) Genome-wide atlas of gene expression in the adult mouse brain. Nature 445: 168–176. 17151600 10.1038/nature05453

[7] Chung SH, Seki K, Choi BI, Kimura KB, Ito A, et al (2010) CXC chemokine receptor 4 expressed in T cells plays an important role in the development of collagen-induced arthritis. Arthritis Res Ther 12: R188. 20939892 10.1186/ar3158 PMC2991023

[8] Takashima Y, Era T, Nakao K, Kondo S, Kasuga M, et al (2007) Neuroepithelial cells supply an initial transient wave of MSC differentiation. Cell 129: 1377–1388. 17604725 10.1016/j.cell.2007.04.028

[9] Furuya S, Makino A, Hirabayashi Y (1998) An improved method for culturing cerebellar Purkinje cells with differentiated dendrites under a mixed monolayer setting. Brain Res Brain Res Protoc 3: 192–198. 9813321 10.1016/s1385-299x(98)00040-3

[10] Farr TD, Liu L, Colwell KL, Whishaw IQ, Metz GA (2006) Bilateral alteration in stepping pattern after unilateral motor cortex injury: a new test strategy for analysis of skilled limb movements in neurological mouse models. J Neurosci Methods 153: 104–113. 16309746 10.1016/j.jneumeth.2005.10.011

[11] Pevny LH, Sockanathan S, Placzek M, Lovell-Badge R (1998) A role for SOX1 in neural determination. Development 125: 1967–1978. 9550729 10.1242/dev.125.10.1967

[12] Li M, Pevny L, Lovell-Badge R, Smith A (1998) Generation of purified neural precursors from embryonic stem cells by lineage selection. Curr Biol 8: 971–974. 9742400 10.1016/s0960-9822(98)70399-9

[13] Klein RS, Rubin JB, Gibson HD, DeHaan EN, Alvarez-Hernandez X, et al (2001) SDF-1 alpha induces chemotaxis and enhances Sonic hedgehog-induced proliferation of cerebellar granule cells. Development 128: 1971–1981. 11493520 10.1242/dev.128.11.1971

[14] Miyata T, Maeda T, Lee JE (1999) NeuroD is required for differentiation of the granule cells in the cerebellum and hippocampus. Genes Dev 13: 1647–1652. 10398678 10.1101/gad.13.13.1647 PMC316850

[15] Bradley P, Berry M (1978) The Purkinje cell dendritic tree in mutant mouse cerebellum. A quantitative Golgi study of Weaver and Staggerer mice. Brain Res 142: 135–141. 75044 10.1016/0006-8993(78)90182-8

[16] Henke RM, Savage TK, Meredith DM, Glasgow SM, Hori K, et al (2009) Neurog2 is a direct downstream target of the Ptf1a-Rbpj transcription complex in dorsal spinal cord. Development 136: 2945–2954. 19641016 10.1242/dev.035352 PMC2723066

[17] Florio M, Leto K, Muzio L, Tinterri A, Badaloni A, et al (2012) Neurogenin 2 regulates progenitor cell-cycle progression and Purkinje cell dendritogenesis in cerebellar development. Development 139: 2308–2320. 22669821 10.1242/dev.075861 PMC3367442

[18] Hoshino M, Nakamura S, Mori K, Kawauchi T, Terao M, et al (2005) Ptf1a, a bHLH transcriptional gene, defines GABAergic neuronal fates in cerebellum. Neuron 47: 201–213. 16039563 10.1016/j.neuron.2005.06.007

[19] Sellick GS, Barker KT, Stolte-Dijkstra I, Fleischmann C, Coleman RJ, et al (2004) Mutations in PTF1A cause pancreatic and cerebellar agenesis. Nat Genet 36: 1301–1305. 15543146 10.1038/ng1475

[20] Al-Shammari M, Al-Husain M, Al-Kharfy T, Alkuraya FS (2011) A novel PTF1A mutation in a patient with severe pancreatic and cerebellar involvement. Clin Genet 80: 196–198. 21749365 10.1111/j.1399-0004.2010.01613.x

[21] Zhu Y, Matsumoto T, Mikami S, Nagasawa T, Murakami F (2009) SDF1/CXCR4 signalling regulates two distinct processes of precerebellar neuronal migration and its depletion leads to abnormal pontine nuclei formation. Development 136: 1919–1928. 19429788 10.1242/dev.032276

[22] Li G, Kataoka H, Coughlin SR, Pleasure SJ (2009) Identification of a transient subpial neurogenic zone in the developing dentate gyrus and its regulation by Cxcl12 and reelin signaling. Development 136: 327–335. 19103804 10.1242/dev.025742 PMC2685973

[23] Lu M, Grove EA, Miller RJ (2002) Abnormal development of the hippocampal dentate gyrus in mice lacking the CXCR4 chemokine receptor. Proc Natl Acad Sci U S A 99: 7090–7095. 11983855 10.1073/pnas.092013799 PMC124533

[24] Lin L, Isacson O (2006) Axonal growth regulation of fetal and embryonic stem cell-derived dopaminergic neurons by Netrin-1 and Slits. Stem Cells 24: 2504–2513. 16840550 10.1634/stemcells.2006-0119 PMC2613222

[25] Marlow R, Strickland P, Lee JS, Wu X, Pebenito M, et al (2008) SLITs suppress tumor growth in vivo by silencing Sdf1/Cxcr4 within breast epithelium. Cancer Res 68: 7819–7827. 18829537 10.1158/0008-5472.CAN-08-1357 PMC3075571

[26] Li X, Lee AY (2010) Semaphorin 5A and plexin-B3 inhibit human glioma cell motility through RhoGDIalpha-mediated inactivation of Rac1 GTPase. J Biol Chem 285: 32436–32445. 20696765 10.1074/jbc.M110.120451 PMC2952245

[27] Pascual M, Abasolo I, Mingorance-Le Meur A, Martinez A, Del Rio JA, et al (2007) Cerebellar GABAergic progenitors adopt an external granule cell-like phenotype in the absence of Ptf1a transcription factor expression. Proc Natl Acad Sci U S A 104: 5193–5198. 17360405 10.1073/pnas.0605699104 PMC1829285

[28] Sidman RL, Lane PW, Dickie MM (1962) Staggerer, a new mutation in the mouse affecting the cerebellum. Science 137: 610–612. 13912552 10.1126/science.137.3530.610

[29] Becker EB, Oliver PL, Glitsch MD, Banks GT, Achilli F, et al (2009) A point mutation in TRPC3 causes abnormal Purkinje cell development and cerebellar ataxia in moonwalker mice. Proc Natl Acad Sci U S A 106: 6706–6711. 19351902 10.1073/pnas.0810599106 PMC2666615

[30] Isaacs AM, Oliver PL, Jones EL, Jeans A, Potter A, et al (2003) A mutation in Af4 is predicted to cause cerebellar ataxia and cataracts in the robotic mouse. J Neurosci 23: 1631–1637. 12629167 10.1523/JNEUROSCI.23-05-01631.2003 PMC6741966

