# Supplementary figures and images for: Ectopic Cerebellar Cell Migration Causes Maldevelopment of Purkinje Cells and Abnormal Motor Behaviour in Cxcr4 Null Mice
Source: PLoS One. 2014 Feb 7;9(2):e86471. doi: 10.1371/journal.pone.0086471 (PMC3917845; doi:10.1371/journal.pone.0086471)

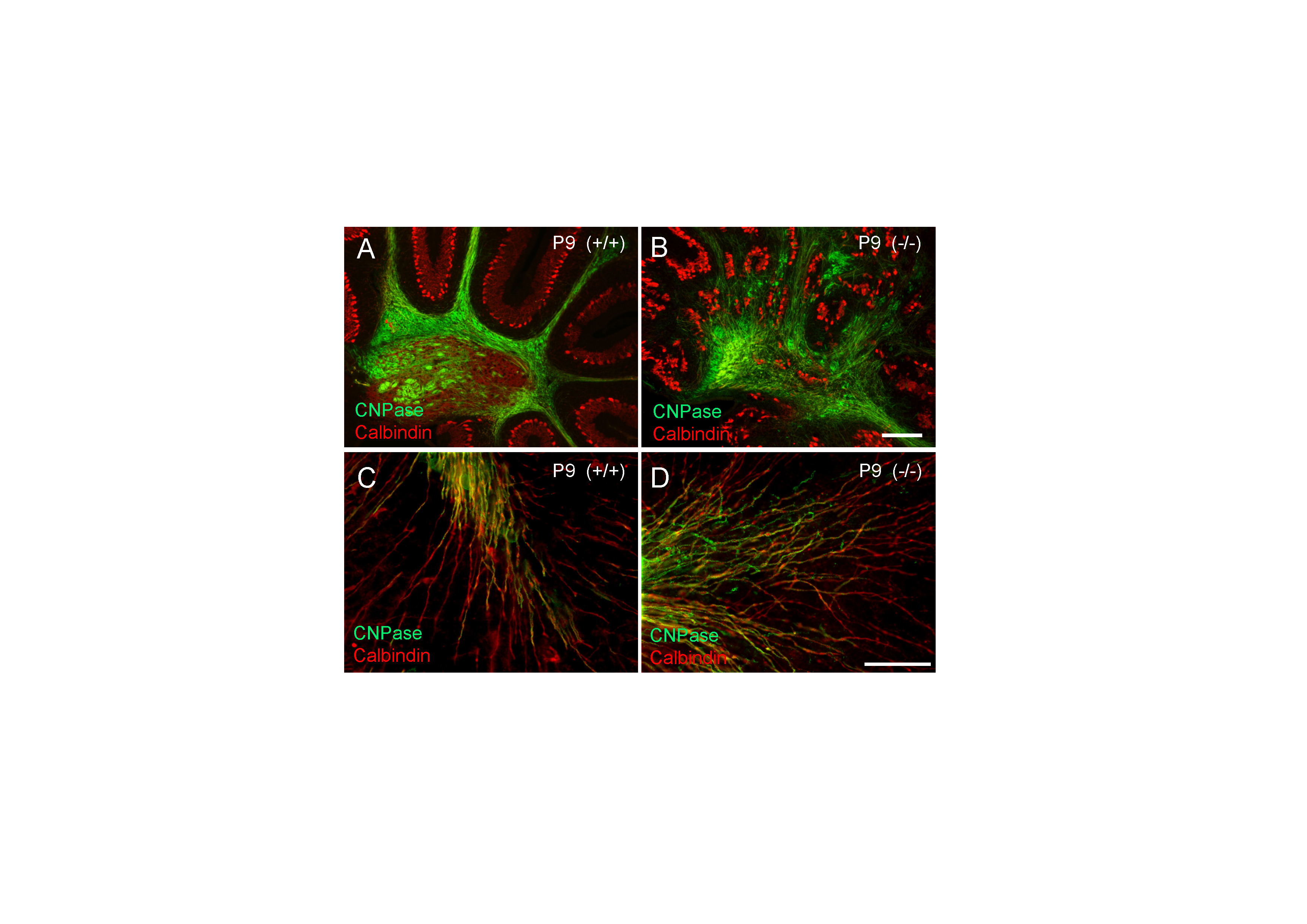

Supplement: Figure S1 — (A,B) The CNPase and Calbindin staining shows that Purkinje cells send axons to deep cerebellar nuclei in both WT and KO mice. (C,D) Greater magnification reveals these Purkinje cell axons are myelinated in both WT and KO mice. Scale bars = 200 µm in A,B; 50 µm in C,D. (TIFF) [file pone.0086471.s001.tiff]

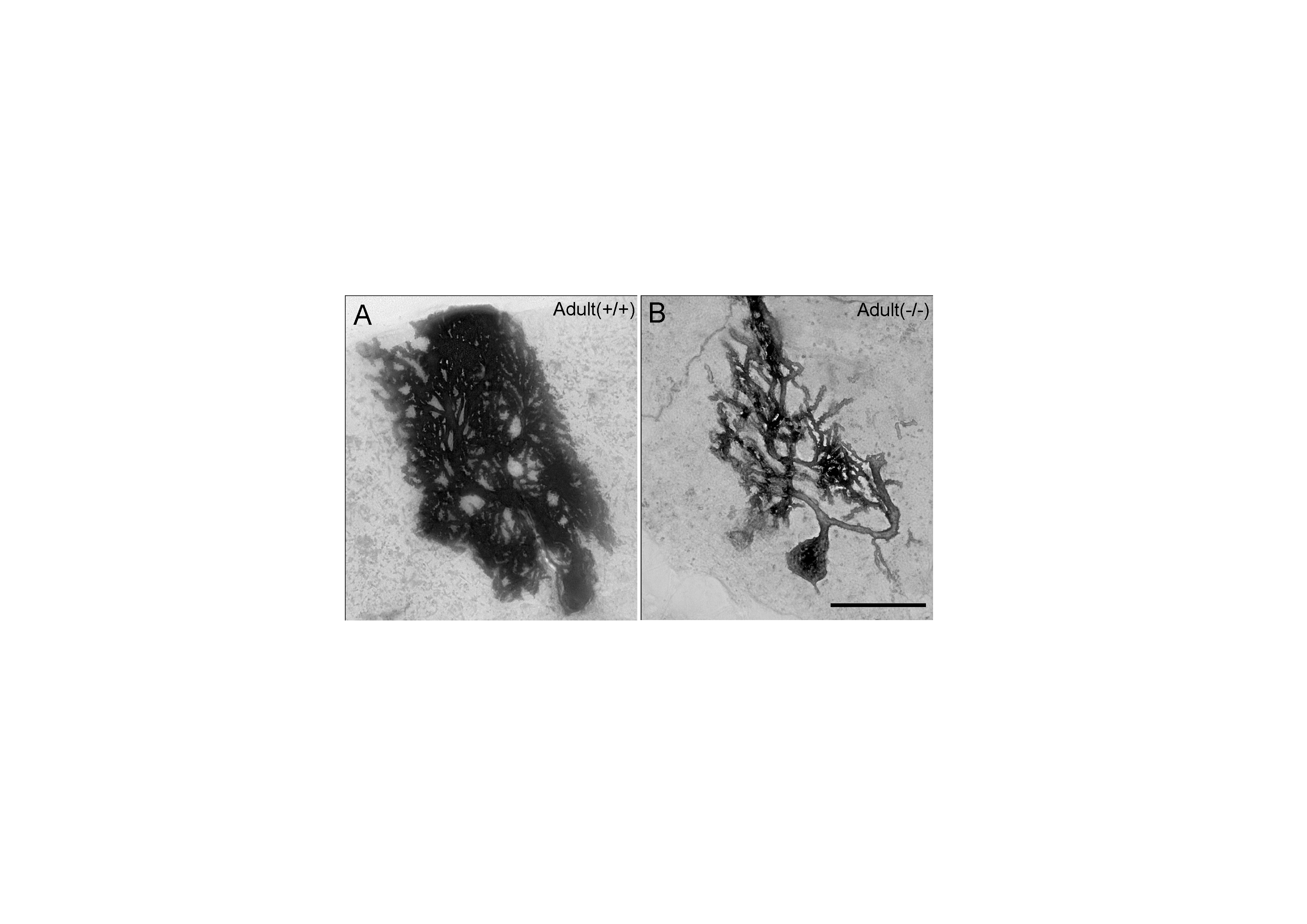

Supplement: Figure S2 — (A,B) Golgi staining showed Purkinje cells in adult KO mice have significant less complex dendritic arborisation. Scale bars = 100 µm. (TIF) [file pone.0086471.s002.tif]
